# The inhibitory effect of some pyrazole ligands and their Cu(II) complexes on the growth of *Escherichia coli, Klebsiella–Enterobacter* spp., and *Staphylococcus aureus*

**DOI:** 10.3389/fphar.2022.921157

**Published:** 2022-08-17

**Authors:** Majda Šahman Zaimović, Milica Kosović Perutović, Gordana Jelušić, Ana Radović, Željko Jaćimović

**Affiliations:** ^1^ Institute for Medicines and Medical Devices of Montenegro, Podgorica, Montenegro; ^2^ University of Montenegro, Faculty of Metallurgy and Technology, Podgorica, Montenegro; ^3^ Moj Lab—Laboratory and Polyclinic, Podgorica, Montenegro; ^4^ Accreditation Body of Montenegro, Podgorica, Montenegro

**Keywords:** pyrazole, antibacterial, *Escherichia*, *Klebsiella*, disc diffusion method

## Abstract

The aim of this study was to evaluate the inhibitory activity of chemistry products against the growth of *Escherichia coli*, *Klebsiella–Enterobacter* spp., and *Staphylococcus aureus*. Pyrazole derivatives (4-bromo-2-(1H-pyrazol-3-yl)phenol, 4-nitro-3-pyrazolecarboxylic acid, N-(benzyloxycarbonyl)-1H-pyrazole-1-carboxamidine), 3-amino-5-hydroxypyrazole, 3,5-pyrazoledicarboxylic acid monohydrate, and selected complexes of Cu(II) with the mentioned pyrazoles as ligands were used as chemistry bioactives for antibacterial activity. The testing method was carried out according to the disc diffusion method. Some compounds have shown inhibitory effects against the growth of *E. coli*. A small number of compounds have shown inhibitory effects against the growth of *Klebsiella–Enterobacter* spp. but did not show inhibitory effects on *Staphylococcus aureus* compared to amoxicillin as a standard.

## Introduction

The resistance to antibacterial drugs has become one of the most important health worries in the last two decades. Infectious diseases caused by antibiotic-resistant bacteria need prerequisite higher doses of drugs, additional lethal treatments, and extended hospital stays [1]. Bacterial pathogens such as *Escherichia coli*, *Staphylococcus aureus*, and *Klebsiella–Enterobacter* spp. are responsible for most drug-resistant infection. These microorganisms cause various infections that range from topical to life-threatening and represent a major problem in hospital and community settings. Among all the infections encountered in hospitals, urinary tract infections are one of the most common infections, in which *E. coli* is found to be the major cause. Several scientific reports have indicated that antibiotic-resistant *E. coli* bacteria are found in soil, water, food, and animals (Chen et al., 2020). *S. aureus* has rapidly acquired resistance to many antibiotic drug classes, causing infections that are difficult to eradicate (Vollaro et al., 2020). *Enterobacter spp*. is a gram-negative environmental bacterium, which belongs to the Enterobacteriaceae family and is found in water, sewage, soil, and plants. These bacteria are common among humans and animals.

Pyrazole derivatives have interesting biological activities such as tumor cell growth activation, antileishmanial activity, anti-inflammatory agents, analgesic activity, antimicrobial activity, anti-inflammatory activity, α-glycosidase inhibitory, antibacterial activity, antioxidant activity, and anticancer agents (Rodhan* et al., 2021; Bhunia et al., 2020; Zebbiche et al., 2022). Many derivatives containing pyrazole nuclei have been commercialized as herbicides, insecticides, and fungicides for plant protection (Abdel Reheim and Baker, 2017).

In the last few years, our group has been continuously working on the synthesis of new pyrazole complexes and determining their antifungal activity (ŽeljkoJaćimović et al., 2018; Latinović et al., 2021). Many pyrazole derivatives and their transition metal complexes have shown significant inhibition of the pathogenic fungi. Some of them have shown 100% efficacy (the patent belongs to the field of conservation of human and animal or plant organisms or their parts or pesticides) [10]. We also examined the Cu(II) complexes with pyrazole derivatives for their antiproliferative activity *in vitro* against four human cancer cell lines: MCF-7 (human breast cancer cell line), T-24 (bladder cancer cell line), A-549 (non–small cell lung cancer), and L-929 (fibroblast-like cell line cloned from strain L) (Galani et al., 2008).

Since most of the pyrazole compounds show antibacterial activity, the synthesized complexes with pyrazoles as ligands are also expected to show antibacterial activity. This study investigated the antibacterial effects of pyrazoles and their complexes in relation to three ATCC (American Type Culture Collection) bacterial species on agar by applying the disc diffusion method. The activity of selected Cu(II) complex with 4-nitro-3-pyrazolecarboxylic acid, Cu(II) complex with N-(benzyloxycarbonyl)-1H-pyrazole-1-carboxamidine, and two Cu(II) complexes with 3,5-pyrazoledicarboxylic acid monohydrate was examined to the mycelial growth of *Escherichia coli*, *Klebsiella–Enterobacter* spp., and *Staphylococcus aureus*. Ligands: 4-bromo-2-(1H-pyrazol-3-yl)phenol (Pz1), 4-nitro-3-pyrazolecarboxylic acid (Pz2), N-(benzyloxycarbonyl)-1H-pyrazole-1-carboxamidine (Pz3), 3-amino-5-hydroxypyrazole (Pz4), and 3,5-pyrazoledicarboxylic acid monohydrate (Pz5) were also used to study the inhibition of mentioned bacteria under laboratory conditions.

## Results

### Antimicrobial effect of chemistry products on *Escherichia coli*

To study the antimicrobial efficiency, two concentrations of Pz1, Pz2, Pz3, Pz4, Pz5, Cu(Pz2)_2_.8H_2_O, [Cu(Pz3)_2_ (MeOH)_2_], {Cu(Pz5) (H_2_O)_2_ (MEOH)_2_}_2_, and Cu(Pz5)_2_ (MeOH)_2_ were added to cultures of *E. coli* and compared with amoxicillin (Table 1). Pz1, Pz2, Pz3, and Cu(II) complexes with Pz2 and Pz3 exhibited high activity against Gram-negative *E. coli* compared with standard amoxicillin. Mean inhibition zones are shown in Tables 1, 2.

**TABLE 1:** Inhibition zones (presented in millimeters) of Pz1, Pz2, Pz3, Pz4, Pz5, and amoxicillin.

*Escherichia*	Amoxicillin	Pz1		Pz2		Pz3		Pz4		Pz5	
		10^−3^	10^−5^	10^−3^	10^−5^	10^−3^	10^−5^	10^−3^	10^−5^	10^−3^	10^−5^
	16.0	10.0	10.0	9.0	9.7	11.4	11.4	0.0	0.0	0.0	0.0
	16.0	9.9	10.0	9.2	9.7	11.4	11.3	0.0	0.0	0.0	0.0
	16.0	9.8	9.9	9.1	9.6	11.2	11.2	0.0	0.0	0.0	0.0
	16.0	10.1	9.8	8.8	9.8	11.4	11.4	0.0	0.0	0.0	0.0
Μ	16.0	10.0	9.9	9.0	9.7	11.4	11.3	0.0	0.0	0.0	0.0

Lsd_00.01_=0.971

**TABLE 2:** Inhibition zones (presented in millimeters) of Cu(Pz2)_2_.8H_2_O (CuPz2), [Cu(Pz3)_2_ (MeOH)_2_] (CuPz3), {C_U_(Pz5) (H2O)_2_ (MEOH)_2_}_2_ (CuPz5_I_), Cu(Pz5)_2_ (MeOH)_2_ (CuPz5_II_), and amoxicillin shown on ATCC strains of *Escherichia coli*.

Escherichia	Amoxicillin	CuPz2		CuPz3		CuPz5i		CuPz5ii	
		10^−3^	10^−5^	10^−3^	10^−5^	10^−3^	10^−5^	10^−3^	10^−5^
	16.0	10.0	11.3	9.7	11.8	0.0	0.0	0.0	0.0
	16.0	9.9	11.3	9.7	11.7	0.0	0.0	0.0	0.0
	16.0	9.9	11.3	9.7	11.6	0.0	0.0	0.0	0.0
	16.0	10.0	11.2	9.8	11.8	0.0	0.0	0.0	0.0
mean	16.0	10.0	11.3	9.7	11.7	0.0	0.0	0.0	0.0

Lsd00.01=0.971

### Antimicrobial effect of chemistry products on *Klebsiella–Enterobacter* spp*.*

To study the antimicrobial efficiency, two concentrations of Pz1, Pz2, Pz3, Pz4, Pz5, Cu(Pz2)_2_.8H_2_O, [Cu(Pz3)_2_ (MeOH)_2_], {Cu(Pz5) (H_2_O)_2_ (MEOH)_2_}_2_, and Cu(Pz5)_2_ (MeOH)_2_ were added to cultures of *Klebsiella–Enterobacter* spp*.* and compared with amoxicillin. Pyrazole derivatives Pz1, Pz2, and Pz3 are highly active against *Klebsiella–Enterobacter*, while other compounds did not cause growth inhibition of these bacteria. Inhibition zones of pyrazole derivatives are shown in Table 3.

**TABLE 3:** Inhibition zones (presented in millimeters) of Pz1, Pz2, Pz3, Pz4, Pz5, and amoxicillin shown on ATCC strains of *Klebsiella*–*Enterobacter* spp.

Klebsiella-Enterobacter	Amoxicillin	Pzl		Pz2		Pz3		Pz4		Pz5	
		10^−3^	10^−5^	10^−3^	10^−5^	10^−3^	10^−5^	10^−3^	10^−5^	10^−3^	10^−5^
	18.0	12.6	13.0	13.6	0.0	13.0	15.0	0.0	0.0	0.0	0.0
	17.9	12.4	13.0	13.5	0.0	13.0	14.8	0.0	0.0	0.0	0.0
	18.0	12.6	12.8	13.5	0.0	13.1	14.9	0.0	0.0	0.0	0.0
	18.0	12.5	9.8	13.6	0.0	13.4	14.7	0.0	0.0	0.0	0.0
mean	18.0	12.5	12.2	13.6	0.0	13.1	14.9	0.0	0.0	0.0	0.0

Lsd_00.01_=0.971

Complexes Cu(Pz2)_2_.8H_2_O (CuPz2), [Cu(Pz3)_2_ (MeOH)_2_] (CuPz3), {C_U_(Pz5) (H_2_O)_2_ (MEOH)_2_}_2_ (CuPz5_I_), and Cu(Pz5)_2_ (MeOH)_2_ (CuPz5_II_) did not show an inhibitory effect on *Klebsiella–Enterobacter* spp.

### Antimicrobial effect of chemistry products on *Staphylococcus aureus*

The used chemical supplements did not cause growth inhibition of *Staphylococcus aureus*, while the disc with amoxicillin showed an inhibition zone of 18 mm.

## Discussion

**FIGURE 1:**
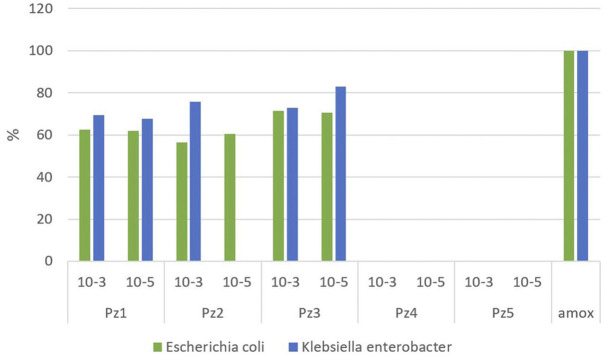
Percentage of inhibition achieved by the pyrazole derivatives in the experiment compared to amoxicillin.

The average percentage of inhibition achieved by pyrazole derivatives used in comparison to control is presented in Figure 1. The pyrazole of formula N-(benzyloxycarbonyl)-1H-pyrazole-1-carboxamidine (Pz3) achieved the best results among other studied compounds at a concentration of 10^−5^ mol/dm^3^ when it inhibited the growth of *Klebsiella–Enterobacter* at a level of 82.8%. This pyrazole showed good results (72.8%) even with a lower concentration of 10^−3^ mol/dm^3^. On the other hand, dilution of the Pz2 solution had a major effect on the inhibition of *Klebsiella–Enterobacter* spp. A solution of pyrazole Pz2 of concentration 10^−3^ mol/dm^3^ gave fairly good results with a percentage of inhibition of 75.6%, while a solution of concentration of 10^−5^ mol/dm^3^ did not show inhibitory activity for these bacteria.

**FIGURE 2:**
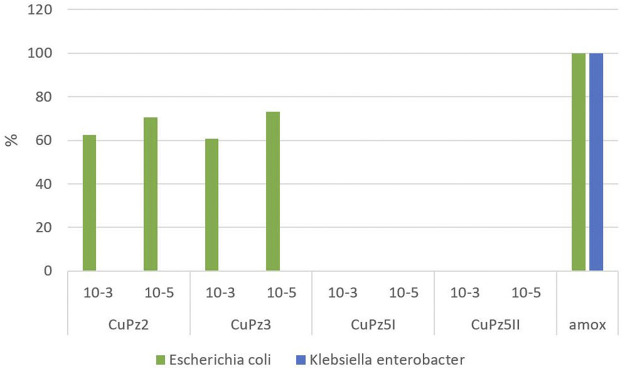
Percentage of inhibition achieved by testing Cu(II) complexes in the experiment compared to amoxicillin.

Among the series, the [Cu(Pz3)_2_ (MeOH)_2_] (CuPz3) compound (10^−5^ mol/dm^3^) was found to be the most active (73.1%) against *E. coli* compared with standard amoxicillin, whereas the same compound did not show any antibacterial activity for *Klebsiella–Enterobacter* spp. [C_U_(Pz5) (H_2_O)_2_ (MEOH)_2_]_2_ (CuPz5_I_) and [Cu(Pz5)_2_ (MeOH)_2_ ](CuPz5_II_) did not show an inhibitory effect on the tested bacteria. The average percentage of inhibition achieved by testing Cu(II) complexes used in comparison to control is presented in Figure 2.

### Tautomeric forms of 3-amino-5-hydroxypyrazole (Pz4)

**FIGURE 3:**
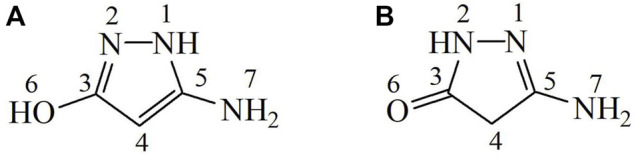
Two tautomeric forms **(A,B)** of the 3-amino-5-hydroxypyrazole (Pz4).

Structural studies of the 3-amino-5-hydroxypyrazole (Pz4) ligand showed that two tautomeric forms A and B are present in the crystal, as shown in Figure 3, where tautomer A is present in a smaller amount than tautomer B.

#### Tautomer A (minor abundance)

^1^H NMR (500 MHz, DMSO-d_6_): δ 4.20 (s, H^4^, 1H), 5.64 (s, H^7^, 2H), and 8.81 (br. s., H^1^+H^6^, 2H).

^13^C{^1^H} NMR (125MHz, DMSO-d_6_): δ 74.23 (C^4^), 159.19 (C^3^ or C^5^), and 170.72 (C^3^ or C^5^).

#### Tautomer B (major abundance)

^1^H NMR (500 MHz, DMSO-d_6_): δ 3.07 (s, H^4^, 2H), 5.85 (s, H^7^, 2H), and 9.83 (s, H^2^, 1H).

^13^C{^1^H} NMR (125 MHz, DMSO-d_6_): δ 36.26 (C^4^), 156.98 (C^5^), and 171.69 (C^3^).

1H (500 MHz), ^13^C (125 MHz), and 15N (50 MHz) NMR spectra were measured on a Bruker spectrometer.

The number of signals in ^13^C{1H} NMR of this compound (five) at 36.26, 74.23, 156.98, 159.19, and 171.69 ppm indicates the presence of two tautomers in DMSO-d6 (three carbon resonances for each tautomer). The sixth signal was observed in the ^13^C, ^1^H NMR spectrum *via* long-range coupling at 170.72 ppm. In line with this spectrum, six resonances were found in the ^1^H NMR spectrum of the compound at 3.07, 4.20, 5.64, 5.85, 8.81, and 9.83 with relative intensities 2:0.3:0.6:2:0.6:1, correspondingly. The spectra measured immediately after dissolution and standing at room temperature for 3 days were identical.

In the ^1^H,^13^C COSY (HSQC) diagram, only two cross-peaks were observed, indicating that the most abundant tautomer contains only one CH_2_ group, whereas the second tautomer contains only one −CH = group. In addition, this implies that the relative abundance of the tautomers is 3:1.

In the ^1^H,^15^N diagram, there were three correlation peaks between ^1^H resonances at 5.64, 5.85, and 9.83 ppm (relative intensities 0.6:2:1) and ^15^N signals at 32.2 ppm (NH_2_ group of tautomer A) and at 41.5 and 145 ppm (NH_2_ and NH groups of tautomer B).

**FIGURE 4:**
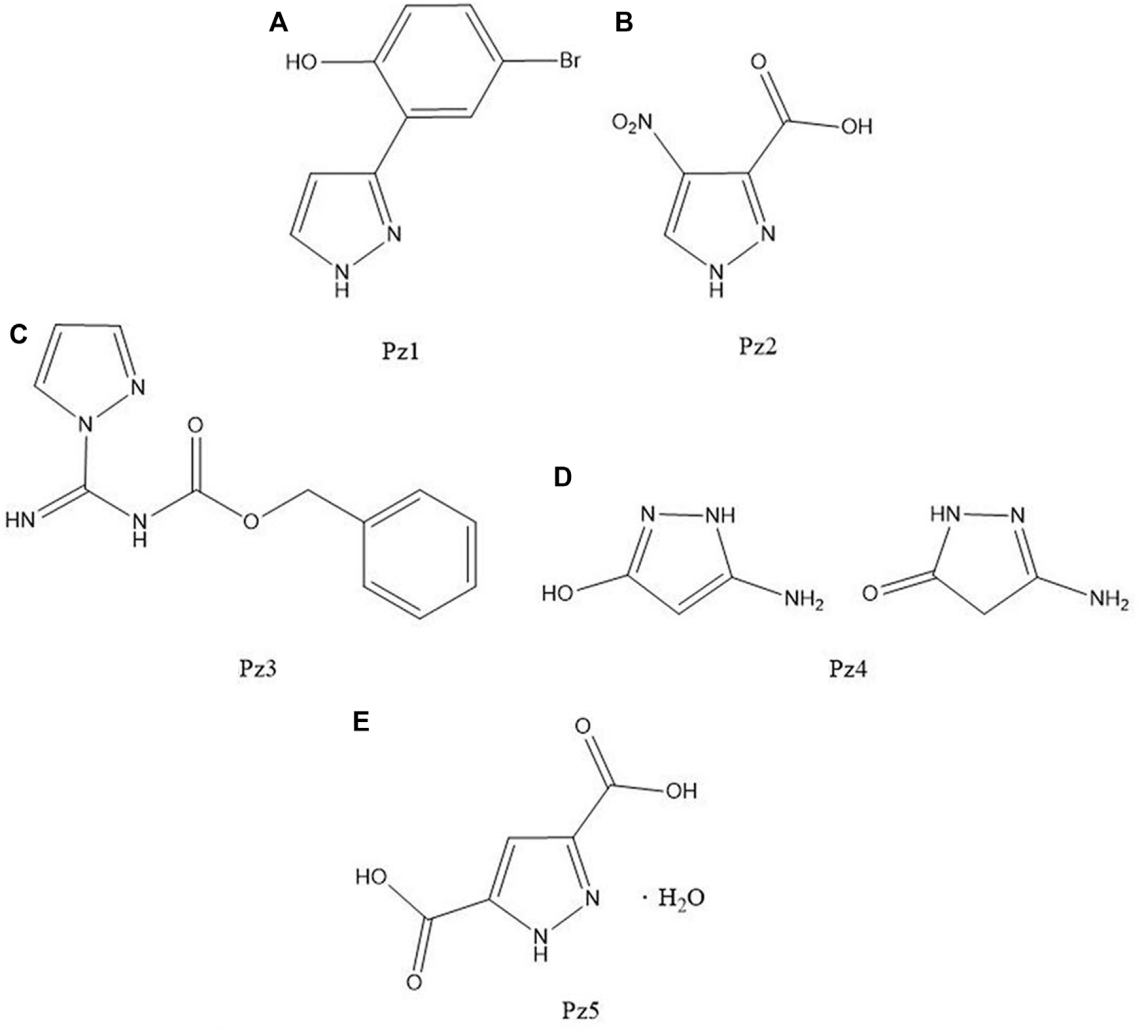
Structure of **(A)** 4-bromo-2-(1H-pyrazol-3-yl)phenol, **(B)** 4-nitro-3-pyrazolecarboxylic acid, **(C)** N-(benzyloxycarbonyl)-1H-pyrazole-1-carboxamidine, **(D)** 3-amino-5-hydroxypyrazole, and **(E)** 3,5-pyrazoledicarboxylic acid monohydrate.

In the long-range ^1^H,^13^C coupling (HMBC) diagram (Figure 4), there were eight cross-peaks for tautomer B—C^4^−H^2^ (3), C^4^−H^7^ (3), C^3^−H^4^ (2), C^5^−H^4^ (2), C^3^−H^7^ (4), C^5^−H^7^ (2), C^3^−H^2^ (2), and C^5^−H^2^ (3) —and three peaks for tautomer A—C^4^−H^7^ (3), C^3^−H^4^ 2), and C^5^−H^4^ (2). This enabled the identification of ^13^C resonances as follows:

Tautomer A—C^3^ and C^5^ at 159.19 and 170.72 ppm;

Tautomer B—C^3^ and C^5^ at 156.98 and 171.68 ppm; the quaternary carbon at 171.68 ppm can presumably be assigned to the C^3^ atom of the carbonyl group.

## Materials and methods

### Pyrazole derivatives

4-Bromo-2-(1H-pyrazol-3-yl)phenol (Pz1), 4-nitro-3-pyrazolecarboxylic acid (Pz2), N-(benzyloxycarbonyl)-1H-pyrazole-1-carboxamidine (Pz3), 3-amino-5-hydroxypyrazole (Pz4), and 3,5-pyrazoledicarboxylic acid monohydrate (Pz5) were commercial products purchased from Sigma-Aldrich Co.

### Synthesis of Cu(II) complexes

**FIGURE 5:**
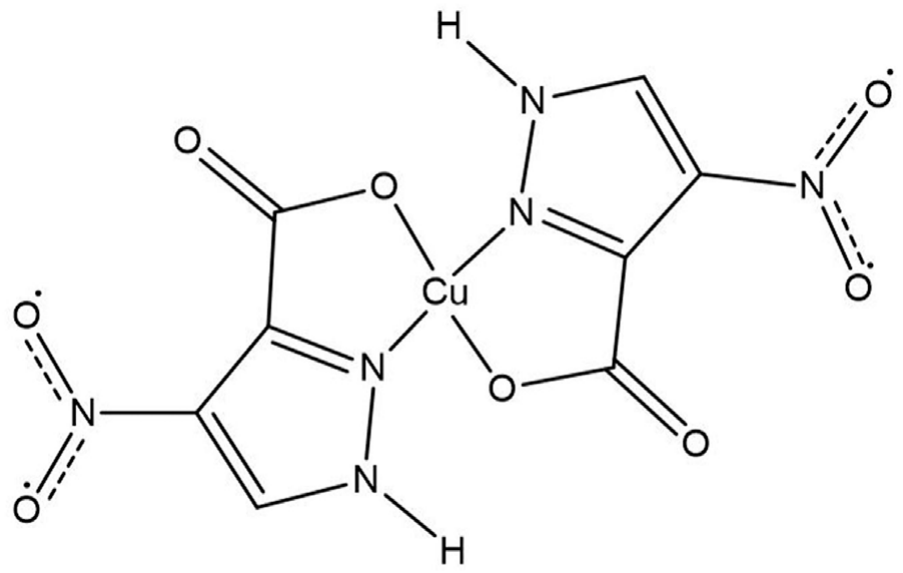
Coordination of Pz2 to Cu(II).

The Cu(II) complex with Pz2 as a ligand was prepared according to the procedure described in a previous article: in the reaction of the warm ethanolic solutions of CuCl_2_^.^2H_2_O and 4-nitro-3-pyrazolecarboxylic acid ligand (Pz2). The bis(ligand) structure of the Cu(Pz2)_2_.8H_2_O complex (Figure 5) was proposed based on elemental analysis, IR spectrometry, and conductometric and TG-MS measurements (ŽeljkoJaćimović et al., 2018).

**FIGURE 6:**
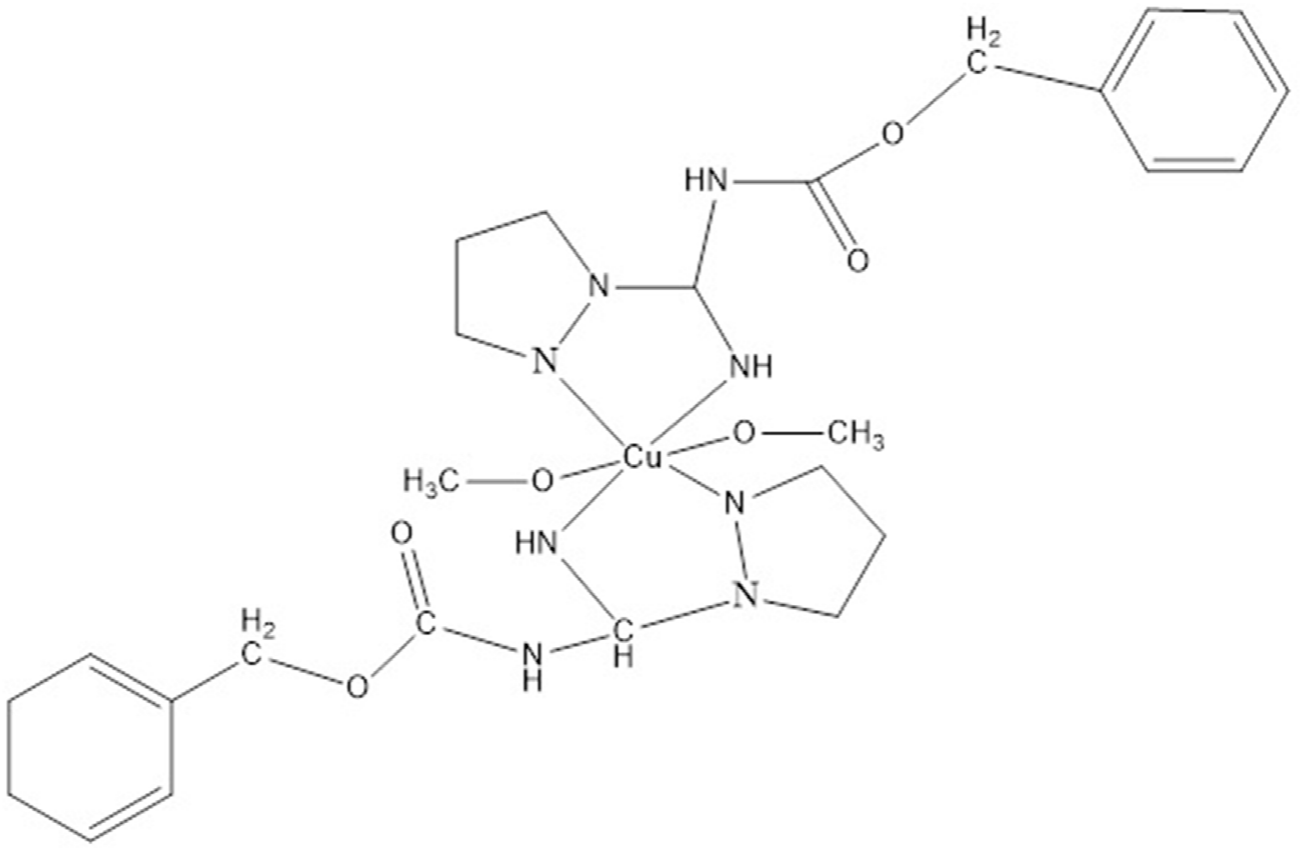
Structure of [Cu(Pz3)2 (MeOH)2].

The [Cu(Pz3)_2_ (MeOH)_2_] complex was obtained by the reaction of methanolic solutions of Cu(OAc)_2_^.^H_2_O and N-(benzyloxycarbonyl)-1H-pyrazole-1-carboxamidine (Pz3). The crystal and molecular structures of the complex (Figure 6) and the ligand (Pz3) were determined by single-crystal X-ray structure analysis (Jaćimović et al., 2017).

Copper complexes with Pz5 and [Cu(Pz5) (H_2_O)_2_ (MEOH)_2_]_2_ were obtained by the next synthesis procedure: 0.25 mmol of copper (II) acetate was dissolved in 0.004 dm^3^ of methanol and mixed with 0.5 mmol of ligand dissolved in 0.004 dm^3^ of methanol with gentle heating. After 24 h, the blue crystals were removed from the mother liquor and transferred to a capillary with methanol without contact with air (when in contact with air, the complex is converted from the monocrystalline form to a light blue powder). The same procedure was used to obtain another complex, [Cu(Pz5)_2_ (MeOH)_2_], with only Cu(II)-nitrate used as a salt. The crystal and molecular structures of the obtained Cu(II) complex compounds were determined by X-ray structural analysis on a single crystal sample.

We used the same quantitative ratios and the same method to obtain the Cu (II) complex with Pz1 and Pz4, but even after several attempts, we could not obtain a suitable product.

### Measurements of Antibacterial Activity

Measurements of Pz1, Pz2, Pz3, Pz4, Pz5, Cu(Pz2)_2_ 8H_2_O, [Cu(Pz3)_2_ (MeOH)_2_], {Cu(Pz5) (H_2_O)_2_ (MEOH)_2_}_2_, and Cu(Pz5)_2_ (MeOH)_2_ for antibacterial activity against *Escherichia coli* (ATCC 25922), *Klebsiella*–*Enterobacter* spp*.* (ATCC 25923), and *Staphylococcus aureus (ATCC 13048)* were carried out using the disc diffusion method. Bacterial strains were obtained from the American Type Culture Collection (ATCC).

Data obtained by measuring the distance between the disc and the bacterial culture are statistically analyzed by analysis of variance, and mean values were compared using the LSD test. If their difference was greater than the LSD test, they were considered statistically significant (Stankovic et al., 1990).

The bacteria were grown on the nonselective media KA-blood agar-finished media (composition: blood agar base plus sheep whole blood; ISO 12485:2003, PROREADY-ProMedia) and selective media EA-Endo agar-finished media (composition: peptone, lactose, sodium sulfate, fuchsine, and agar; ISO 12485:2003 PROREADY-ProMedia). The strains were cultivated on KA and EA media after being placed in a saline solution with a density of 0.5 per McFarland standards. The plates were incubated at 37°C for 24 h. After 24 h, we made a dilution of the given cultures: 10^−3^ and 10^−5^ mol/dm^3^.

The ligand/complex was mounted as a paper disc (prepared from blotting paper with the help of a micropipette). After the discs were applied on the previously inoculated agar plates, the plates were incubated at 37°C for 24 h.

At the same time, control discs contained 24 µg/disc amoxicillin for all bacteria. The diameters of the inhibition zones were measured in mm, and their mean values were calculated. Statistical analysis was carried out in relation to reference zones of inhibition by EUCAST (European Committee for Antimicrobial Susceptibility Testing)

## Conclusion

In conclusion, pyrazole derivatives and their Cu(II) complexes were tested for antimicrobial measurements. None of the tested compounds showed an inhibitory effect on *Staphylococcus aureus*. Based on the obtained results, it can be concluded that pyrazoles and the synthesized complexes with pyrazoles as ligands have shown good activity toward *E. coli* compared to the control. In contrast to the tested complex compounds and pyrazole derivatives Pz4 and Pz5, which did not show inhibition for *Klebsiella*–*Enterobacter* spp*.*, antifungal activity of Pz1, Pz2, and Pz3 compounds was greatly active against this bacterium (the inhibition reached 82.8%). The results also showed how a change in the concentration of the same compound affects the inhibition, especially by reducing the concentration of pyrazole. This compound changes from a fairly active to an inactive compound, observing its effect on the bacterium *Klebsiella*–*Enterobacter* spp. Taking all results into consideration, these derivatives could be extremely important molecules for further development of antimicrobial agents.

## Data Availability

The original contributions presented in the study are included in the article/Supplementary Material. Further inquiries can be directed to the corresponding author.
